# The Use of Mobile Phone and Medical Apps among General Practitioners in Hangzhou City, Eastern China

**DOI:** 10.2196/mhealth.4508

**Published:** 2016-05-24

**Authors:** Ying Liu, Wen Ren, Yan Qiu, Juanjuan Liu, Pei Yin, Jingjing Ren

**Affiliations:** ^1^ General Practice Department, The First Affiliated Hospital College of Medicine Zhejiang University Hangzhou China

**Keywords:** mobile phone, app use, general practitioner, mobile technology

## Abstract

**Background:**

Mobile phones and mobile phone apps have expanded new forms of health professionals’ work. There are many studies on the use of mobile phone apps for different specialists. However, there are no studies on the current use of mobile phone apps among general practitioners (GPs).

**Objective:**

The objective of the study was to investigate the extent to which GPs own smartphones with apps and use them to aid their clinical activities.

**Methods:**

A questionnaire survey of GPs was undertaken in Hangzhou, Eastern China. Data probing GPs’ current use of medical apps in their clinical activities and factors influencing app use were collected and analyzed

**Results:**

125 GPs participated in the survey. 90.4% of GPs owned a mobile phone, with 48.7% owning an iPhone and 47.8% owning an Android phone. Most mobile phone owners had 1-3 medical-related apps, with very few owning more than 4. There was no difference in number of apps between iPhone and Android owners (*χ*^2^=1.388, *P*=0.846). 36% of GPs reported using medical-related apps on a daily basis. The majority of doctors reported using apps to aid clinical activities less than 30 minutes per day.

**Conclusions:**

A high level of mobile phone ownership and usage among GPs was found in this study, but few people chose medical-related apps to support their clinical practice.

## Introduction

Mobile phone use has drastically changed the way we work and live. From Internet access to email, it offers on-the-go access to information with unprecedented ease. The ownership of mobile phones by health professionals is increasingly common, and health professionals’ use of mobile phone apps has changed how they do their work [1].

Previous literature has examined mobile phone acceptance and medical app usage with medical students, interns, junior doctors, and trainees. For example, Payne et al [2] found a high degree of mobile phone ownership and use among medical students and junior doctors. They endorsed the development of more apps to support education and clinical practice. One study showed that urology trainees in Ireland found mobile phones to be a useful aid in clinical practice both as an educational and reference tool [3]. Another study revealed that 85% of medical providers working in Accreditation Council for Graduate Medical Education (ACGME) training programs reported use of mobile phones [4]. Mobile phone usage was also widespread among interns in Ireland university hospitals, where apps could aid clinical practice and education [5]. Rung et al studied mobile phone use in Australian dental students, and concluded that students used mobile phones for their education although the technology had not been formally included in the curriculum [6].

Mobile phones with medical apps have been demonstrated to be very useful for medical students and physicians. However, there are no studies on the current use of apps among general practitioners (GPs). GPs, known as the gatekeepers of health care, are doctors who treat acute and chronic illnesses and provide preventive care and health education irrespective of age and sex. Though GPs comprise a small proportion of all doctors in China (5.6%), they readily take on the role of health gatekeepers [7]. GPs who work in community health centers (CHCs) provide *Six in One* services for Chinese residents. *Six in One* services include preventive care, health care, medical care, rehabilitation, health education and family planning technology guidance, and all 6 services are provided by GP-centered primary care teams. Studies showed that mobile phones and apps can be useful for patient education, remote patient monitoring, mobile clinical communication, disease management and so on [8-10], and could thus support general practices. In this study, we conducted a survey on mobile phone usage among GPs in a city located in eastern China.

The objectives of this study were (1) to identify the extent to which GPs own mobile phones and which types, (2) to evaluate how often they use apps to acquire medical information and support clinical decisions, and (3) to investigate the types and frequency of apps used. This study provides a preliminary glimpse into a cross-sectional study on mobile phone ownership and usage among GPs in eastern China.

## Methods

A cross-sectional study was designed and conducted from October to December 2014 in Hangzhou city, the capital of Zhejiang Province, Eastern China. Hangzhou has a population of 8.8 million and is ranked tenth in terms of gross domestic product per capita among China's 100 major cities.

Six CHCs were randomly selected from the six main districts of Hangzhou city. All the GPs who were working in these centers (defined as having had acquired a medical license and finished 1-3 years GP training) completed a questionnaire on paper. The study was approved by the ethics committee of the First Affiliated Hospital, College of Medicine, Zhejiang University.

The questionnaire and definitions of medical apps used in the survey were derived from a similar study conducted in the UK [2]. To ensure the validity of the questionnaire, an expert panel (6 doctors having at least 5 years’ experience in general practice or primary care research) was invited to review and revise it. The questionnaire was then piloted within one CHC and altered accordingly. The final questionnaire was composed of 5 questions probing the GPs’ current use of medical apps in their clinical activities and influencing factors, including: the number who owned a mobile phone; type of mobile phone; the number of medical apps owned; how often medical apps were actually referred to during working hours; and the clinical environment in which non-medical mobile phone apps were used. The number of medical apps they owned was determined by how many of these apps were on their mobile phone during the study. Medical apps were divided into the following categories: literature search tools, disease diagnosis/management apps, medical calculators, and drug reference apps [1-2].

All numerical data were managed and analyzed by the Statistical Package for Social Sciences (SPSS Inc.). Initial descriptive statistics were determined and the Chi-square test was used for inferential analyses.

## Results

A total of 125 GPs working in 6 CHCs participated in the survey; the respondent rate was 100%. The male to female split was 45.6% (57/125) and 54.4% (68/125) respectively. The mean age of participants was 36.2 years (SD 6.9) with a range of 25 to 60 years, while 61 GPs were younger than 35 years. Regarding the educational level, 106 participants had a bachelor degree and above. 118 participants had been involved in the GP training program, while only 26 participants took part in the Medical Information Program.

Regarding working experience, 92 participants had been working in General Practice for more than 5 years. In China, a medical doctor’s professorial equivalent is expressed in 5 levels (from senior to junior), which are: (1) chief doctor (equivalent to professor grade in universities), (2) associate chief doctor (equivalent to associate professor grade in universities), (3) attending specialist (equivalent to lecturer grade in universities), (4) resident (acquired a bachelor degree and works at a stage of postgraduate medical training), and (5) assistant doctor (acquired a three-year medical diploma). With respect to professional grade, 9 participants were chief doctors or associate chief doctors and 78 participants were attending specialists. The monthly income of most GPs (88%, 110/125) varied from US $500 to US $800, while the income range was US $320 to US $1600.

There were 113 participants who owned a mobile phone, 55 with an iPhone, 54 with an Android, and 4 owning other mobile phones. Young participants (under the age of 35) were more likely to own a mobile phone (*χ*^2^=8.705, *P*=0.013). There were no significant association with gender, education, title, time of working in General Practice, income and GP training program.

The number of medical apps installed by GPs is displayed in Table 1. 75 mobile phone owners had medical apps. Among them, 39 were iPhone owners, 39 had Android phones and 4 users had ‘other’ mobile phones. There was no difference between iPhone and Android users who own apps (*χ*^2^=1.4, *P*=0.846). 32 mobile phone users reported downloading 1 medical app on their mobile phone, 21 downloaded 2 apps, 12 downloaded 3 apps, 10 downloaded 4-15, and 38 users reported having no medical apps on their mobile phone.

Table 2 shows the frequency of medical apps used by GPs. Among these 75 mobile phone users who have downloaded medical apps, 27 GPs reported using them on a daily basis, 24 GPs on a weekly basis while 18 reported rarely using them and 6 GPs never used these apps.

Table 3 shows the time GPs used medical apps on a daily basis to aid clinical activities. 27 GPs responded that they used medical apps every day. However, 25 GPs used medical apps to aid clinical activities less than 30 minutes a day.

**Table 1:** Number of medical apps installed by GPs.

		Total (N=113)	iPhone users (n=55)	Android users (n=54)	Others (n=4)
**Own medical apps**					
	No	33.6% (38/113)	29.1% (16/55)	37.0% (20/54)	50.0% (2/4)
	1 app	28.3% (32/113)	32.7% (18/55)	25.9% (14/54)	0 (0)
	2 apps	18.6 % (21/113)	16.4% (9/55)	18.5% (10/54)	50.0% (2/4)
	3 apps	10.6% (12/113)	10.9% (6/55)	11.1% (6/54)	0 (0)
	4-15 apps	8.9% (10/113)	10.9% (6/55)	7.5% (4/54)	0 (0)

**Table 2:** Frequency of medical apps used by GPs.

Frequency of use	Percentage who used (n=75)
Daily	36% (27/75)
Weekly	32% (24/75)
Rarely	24% (18/75)
Never used	8% (6/75)

**Table 3:** Daily use, in minutes, of GPs’ medical app use for clinical activities.

Daily use of apps	Percentage who used (n=27)
None	4% (1/27)
1-10 minutes	26% (7/27)
11-20 minutes	37% (10/27)
21-30 minutes	26% (7/27)
> 31 minutes	7% (2/27)

**Figure 1:**
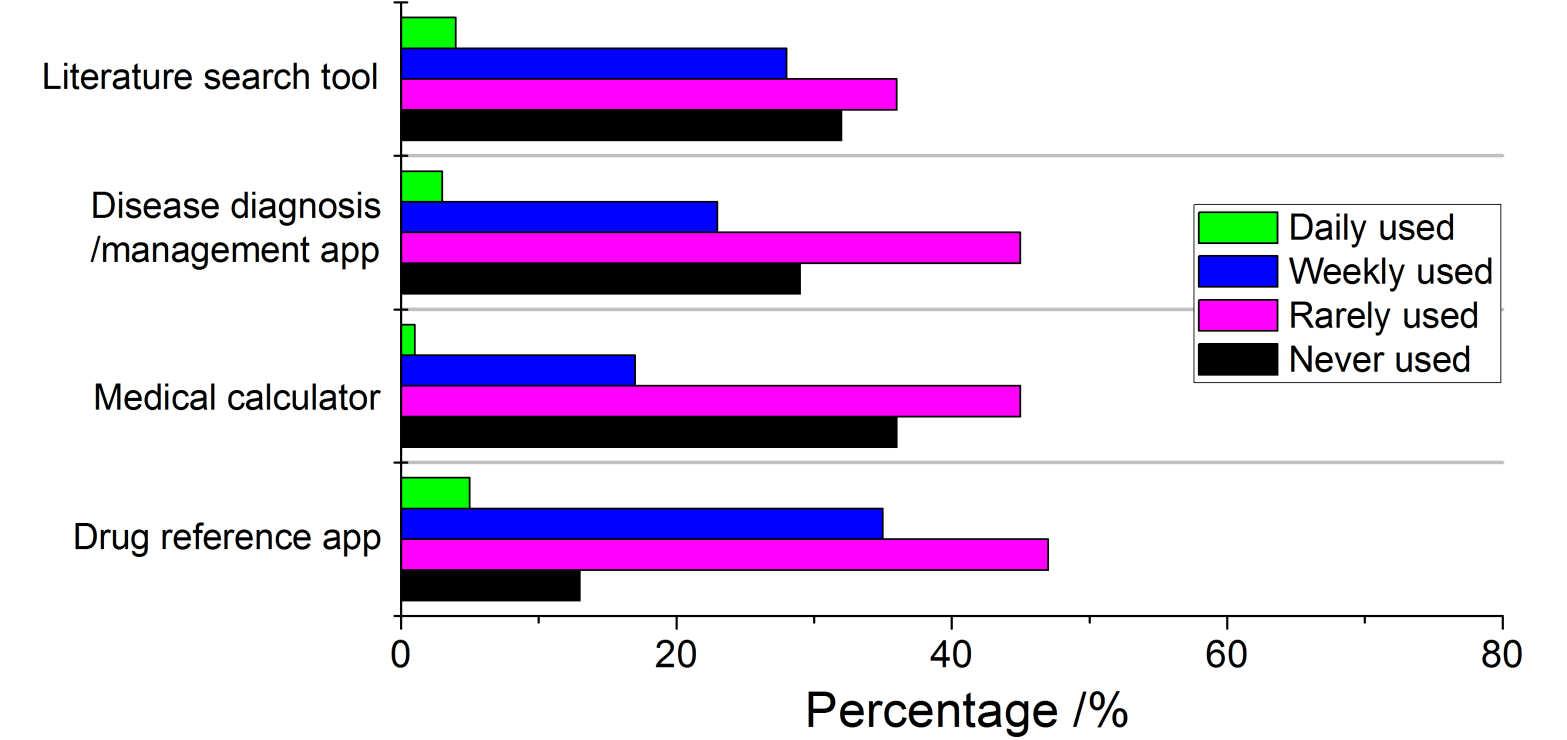
Type of medical app and frequency of use by GPs.

**Figure 2:**
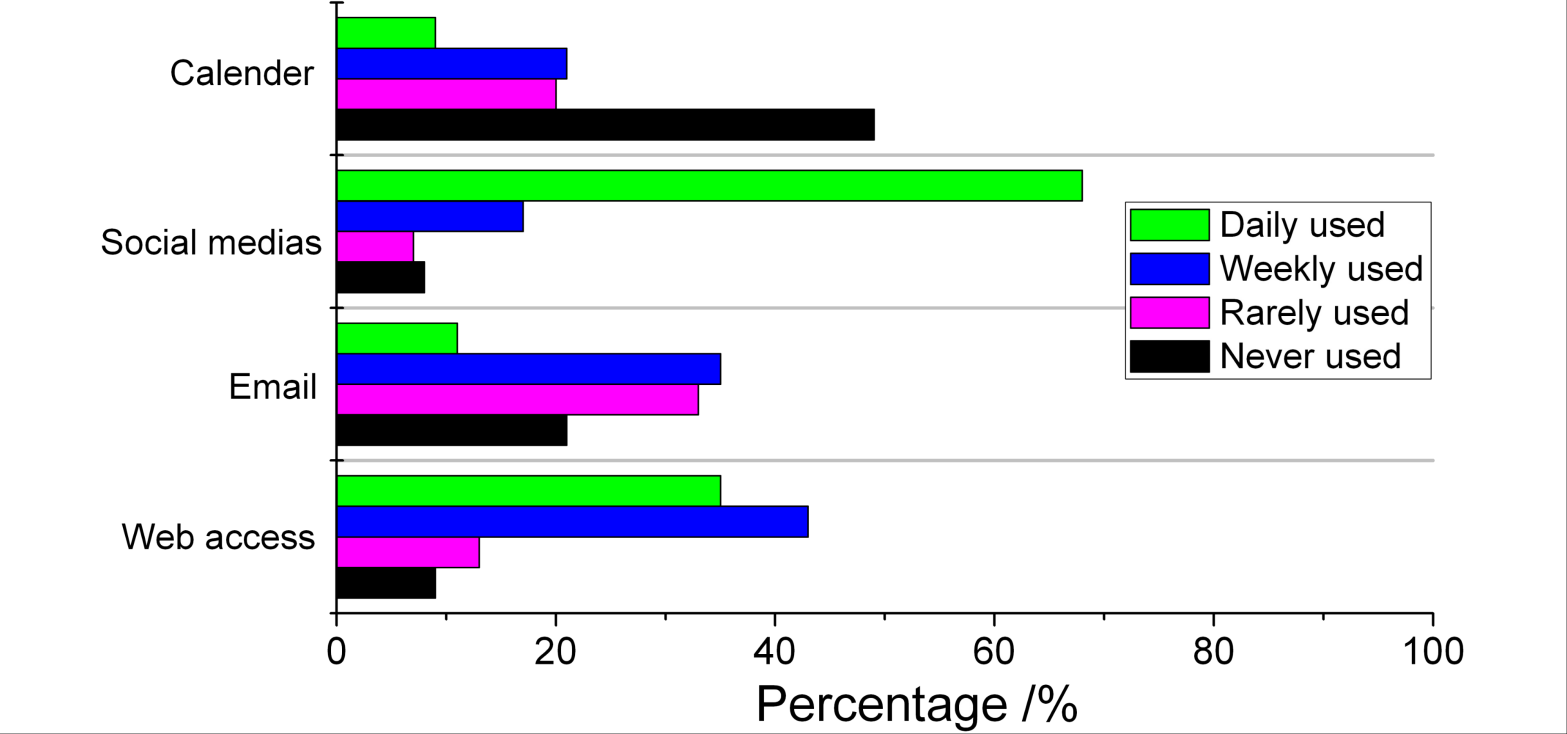
Type of non-medical app and frequency of use by GPs.

Figures 1 and 2 show the type of medical and non-medical apps used by GPs, and how often these were used. According to these two figures, they used non-medical apps much more frequently than medical apps. The most frequent type of medical app used was drug reference apps; non-medical apps were most frequently social media apps.

## Discussion

### Overview

This study is the first formal survey to investigate mobile phone ownership and usage among GPs in China. The result of our study demonstrates that a substantial proportion (90.4%) of GPs use mobile phones, with no difference based on income level or professional grades (Table 1). Previously, in the UK, a regional survey conducted on mobile phone-based healthcare apps usage by Payne et al [2] in 2011 found 74.8% of junior doctors owned a mobile phone (n=601). In the US, a nation-wide email survey of all the ACGME training programs (N=3,306) revealed that 85% of respondents used a mobile phone [4]. In Ireland, a voluntary novel questionnaire survey revealed that, among interns in two university hospitals, 98.4% owned a mobile phone [5]. Though the income for GPs in China is lower than Western countries, the percent of mobile phone ownership is higher than the UK and the US, and lower than Ireland. It should be pointed out that the surveys in UK and US were conducted before 2012, when mobile phones were not so widespread. An investigation from Google and IPSOS in 2013 revealed that mobile phone penetration is currently at 47% of the population in China [11]. Our research shows that mobile phone ownership among GPs in eastern China is higher than that of the general public. The common feature of previously-mentioned studies is that the selected subjects were young doctors, while in this study GPs at every age level were included. The extreme popularity of mobile phones among all ages demonstrates how well-accepted mobile phones are among Chinese GPs. When examining the differences between ages, our survey suggested that young GPs (under 35 years) are more likely to embrace mobile phones, perhaps due to the fact that they are more “technologically adept” [2].

In our survey, 66.4% of mobile phone users reported downloading medical apps, 86.7% of whom own 1-3 such apps (Table 1). In the UK, 75.5% of mobile phone user used medical apps among junior doctors, and the majority (51%) of them owned 1 to 5 medical apps [2]. In the US, 63.5% of doctors used medical apps [4]. In Ireland, 77% of urology trainees [3] and 91.6% of interns [5] downloaded apps. With these intern mobile phone users, 53.3% reported downloading between 1 and 3 medical apps [5]. Research from all these countries show that the majority of doctors download few medical apps, perhaps due to the cost of apps and Internet connections, limited working time, the capacity of their mobile phones, and high workload [2,12].

In Table 2, we see that 36% of GPs report using medical apps on a daily basis. In Table 3, the majority of doctors report using medical apps daily less than 30 minutes to aid the clinical activities. Compared with other surveys, these results are higher than junior doctors in the UK, but lower than interns in Ireland. In the UK, 29.6% of junior doctors reported using apps on a daily basis and most of them using apps less than 30 minutes per day [2]. In Ireland, 43.6 % interns report using them on a daily basis [5]. This suggests that doctors are using apps as a source of quick references [2], and the conclusion could be drawn that convenience and speed are the main reasons for doctors choosing apps.

When we try to investigate which kinds of apps for GPs were used frequently, interestingly, we found that non-medical apps were used much more frequently to aid their medical activity than medical apps during working hours, as shown in Figure 1 and 2. 67% of GPs use social media apps every day to support clinical activities. Most of the clinical activities included reading case-related essays, as reported by the respondents. Web browsers were the second most popular type of app (34% report daily use) which GPs use to search medical knowledge. By contrast, the most frequently used type of medical app–drug reference apps–only have 5% daily users. This remarkable contrast can be attributed to 2 reasons. First of all, the apps’ function cannot meet their needs [4]. Some respondents in our survey told the investigator that using apps to aid clinical activities is waste of time and they did not have time to use them. Secondly, while medical apps have very specialized applications, non-medical apps provide more diverse functions. For instance, social media can provide an online community where doctors can read articles, listen to experts, find new medical research results, and communicate with colleagues and patients [13]. On the other hand, most medical apps have limited function, and as a result, their acceptance is not as high as non-medical apps.

With medical apps, the most frequently used in China are for drug reference, while social media is the most popular type of non-medical app. In contrast, junior doctors in the UK used clinical scoring apps more often [2]. There are two possible explanations for this. First of all, in Chinese hospitals, there are many traditional Chinese medicines, and different hospitals own different medicines from various companies, resulting in a complex pharmaceutical system. Moreover, patients may approach GPs with prescriptions received from other hospitals. As such, GPs have to constantly update their knowledge about different kinds of medicines. Secondly, in China, most GPs take care of their patients based on their own experiences rather than evidence-based medicine, and they rarely use clinical scoring apps to aid their work.

### Limitations

This study only focuses on GPs who are working in CHCs located in one city, and those working in various levels of hospitals are not involved. Thus the study cannot speak to circumstances for GPs in other areas of China. In addition, since no psychometric properties of the questionnaire are explored, the reliability of the instrument is unknown.

### Conclusions

This study shows that mobile phones are popular among GPs in eastern China. Though the frequency of medical app use by GPs is similar with doctors in other countries, few GPs choose medical apps to support their clinical practice. Due to the behavioral habits of these GPs and the narrow usage of specific medical apps, non-medical apps such as social media were more frequently used to aid their medical activity during working hours. Mobile phones can be useful for many aspects of general practice, such as patient care, health education, and professional communication. However, all these functions have not been widely used by Chinese GPs, which needs to be explored in the future; apps especially designed for GPs with these specific functions are expected for future development.

## Abbreviations

ACGME: Accreditation Council for Graduate Medical Education
CHC: community health center
GP: general practitioner
